# Untargeted LC-HRMS Metabolomics for the Detection of Alternaria-Infected Apples Under Retail and Storage Conditions

**DOI:** 10.3390/toxins18040159

**Published:** 2026-03-27

**Authors:** María Agustina Pavicich, Claudia Giménez-Campillo, José Diana Di Mavungu, Sarah De Saeger, Andrea Patriarca

**Affiliations:** 1 Centre of Excellence in Mycotoxicology and Public Health, Department of Bioanalysis, Faculty of Pharmaceutical Sciences, Ghent University, 9000 Ghent, Belgium; jose.dianadimavungu@ugent.be (J.D.D.M.); sarah.desaeger@ugent.be (S.D.S.); 2 Laboratorio de Microbiología de Alimentos, Departamento de Química Orgánica, Facultad de Ciencias Exactas y Naturales, CONICET, Instituto de Micología y Botánica (INMIBO), Universidad de Buenos Aires, Buenos Aires C1428EGA, Argentina; andrea.patriarca@cranfield.ac.uk; 3 Department of Analytical Chemistry, Faculty of Chemistry, University of Murcia, Regional Campus of International Excellence “Campus Mare Nostrum”, E-30100 Murcia, Spain; claudia.gimenez@um.es; 4 Laboratory of Integrative Metabolomics, Department of Translational Physiology, Infectiology and Public Health, Faculty of Veterinary Medicine, Ghent University, 9820 Merelbeke, Belgium; 5 Department of Biotechnology and Food Technology, Faculty of Science, Doornfontein Campus, University of Johannesburg, Gauteng 2028, South Africa; 6 Magan Centre of Applied Mycology, Faculty of Engineering and Applied Sciences, Cranfield University, College Road, Bedford MK43 0AL, UK

**Keywords:** mycotoxins, food safety, chemometrics

## Abstract

Apples are highly susceptible to fungal infections, particularly by *Alternaria* species, which can lead to fruit deterioration and mycotoxin contamination during storage. This study aimed to evaluate the potential of untargeted liquid chromatography–high-resolution mass spectrometry (LC-HRMS) as a control-oriented strategy to detect *Alternaria*-infected apples under retail and long-term storage conditions. Healthy Red Delicious apples were artificially inoculated with three *Alternaria tenuissima* strains on the fruit surface or core and incubated at 25 °C or 4 °C. Extracts were analysed by UPLC-HRMS in both positive and negative electrospray ionisation modes, followed by multivariate chemometric analysis. Principal component analysis and partial least squares discriminant analysis consistently discriminated infected from non-infected apples, independent of strain, infection site, or incubation temperature. Feature selection based on variable importance values significantly improved model robustness and predictive performance. The metabolomic profiles also enabled discrimination according to *Alternaria* strain, infection site, storage temperature, and selected combinations of these factors. The results demonstrate that LC-HRMS-based untargeted metabolomics could provide a statistically robust framework for detecting *Alternaria tenuissima* infection in apples under the studied conditions.

## 1. Introduction

Apples are one of the most important fruits worldwide, with an annual production of 97 million tonnes [1]. Both the fresh fruit and its numerous food products (juice, purées, compote, and dried apples) are consumed in a wide variety of countries, due to their high nutritional value and pleasant taste [2]. This fruit is highly vulnerable to fungal infections throughout the various stages of production, from cultivation and harvesting to transport and storage, with a particularly high incidence of rotting during the post-harvest period [3]. Several species of the genus *Alternaria* are considered among the most relevant pathogens in the post-harvest stage of apples, with infection rates reaching up to 41% in commercial fruits of the Red Delicious variety in Argentina [4]. The development of these fungi in fresh apples is a major concern, not only because of the deterioration of the fruit, but also because of their ability to produce mycotoxins [5]. This pathogen not only infects the fruit on the exterior; mouldy core (MC) is also a frequent disease that favours mycotoxin accumulation even at refrigeration temperatures inside the fruit, usually in the seed and carpel wall. In a previous study [6], the legislation on *Alternaria* mycotoxins in apple by-products was suggested since a risk for consumers was demonstrated, particularly for the vulnerable group of children. Nonetheless, legislation alone does not prevent the accumulation of toxins; controls in the process of apple by-products are necessary for producers to be able to comply with future legislation.

The incidence of *Alternaria* increases during the post-harvest stage [4]. Thus, storing fruits for shorter periods and processing freshly harvested apples would be an effective strategy for reducing mycotoxin concentration in the final products, but it is not always possible or profitable. Recent advances in mass spectrometry-based analytical strategies have expanded the applications of both untargeted and targeted metabolomics in food and agricultural research. Targeted approaches allow sensitive and quantitative determination of predetermined metabolites relevant to contamination, whereas untargeted fingerprinting enables the detection of unknown markers and broad biochemical changes associated with food quality and safety risks. These complementary strategies have been successfully applied to identify biomarkers, detect adulterants, and assess secondary metabolites that may pose food safety concerns [7]. The application of untargeted metabolomics towards food safety strategies is still in early stages, but the work done to date shows promising results. These studies employ various analytical hardware and software—many using high-resolution mass spectrometry (HRMS) [8]—and enable the identification of unique metabolic markers that can be applied in the early detection of a phytopathogen or its metabolites as a fingerprint of infection [9]. Therefore, untargeted metabolomics can be used to develop strategies to tackle the issue of the contamination of food and feed with mycotoxins through an understanding of the plant–pathogen interaction [10,11].

The aim of this study was to set the basis for a control strategy based on liquid chromatography tandem HRMS to detect apples infected with *Alternaria tenuissima* through metabolomic signatures associated with fungal infection and potential mycotoxin production. To this end, untargeted HRMS analyses were carried out in positive and negative ionisation modes and combined with chemometric tools to discriminate between infected and uninfected apples in a single analysis. The ability of the proposed approach to differentiate between different strains, storage conditions (temperature) and the area of the fruit affected was also evaluated by analysing the individual effects of these factors and their possible interactions.

## 2. Results

Composite ion maps which contained 21,804 and 21,506 metabolomic features in ESI^+^ and ESI^−^ mode, respectively, were obtained. Filtering of the features according to the ANOVA *p*-value < 0.05 decreased the number to 9308 and 10,505 metabolites in ESI^+^ and ESI^−^, respectively. Supplementary Tables S1 and S2 present all selected features used for downstream analysis in ESI^+^ and ESI^−^ modes, respectively. This data reduction allowed the study to focus on features that clearly discriminate infected from non-infected apples by principal component analysis (PCA).

**Figure 1:**
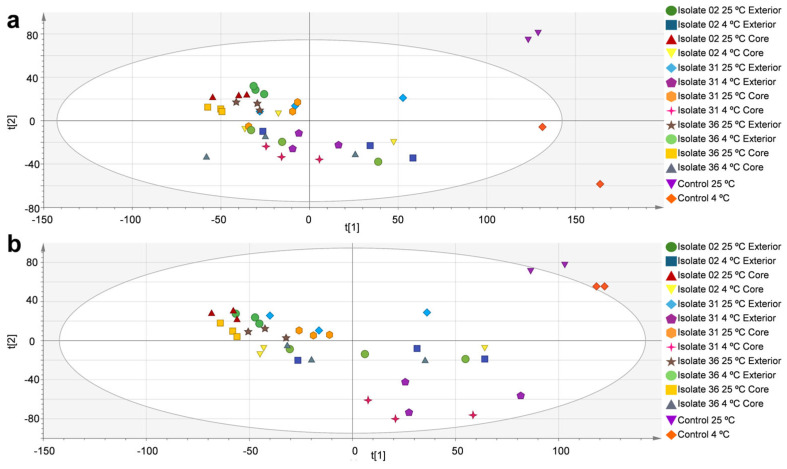
Principal component analysis of metabolites in healthy apples and apples inoculated with *Alternaria tenuissima* under the different incubation conditions. (**a**) ESI^+^ mode (R2X = 0.684 and Q2 = 0.379) and (**b**) ESI^−^ mode (R2X = 0.682 and Q2 = 0.382).

Figure 1a shows the PCA of metabolomic features detected in infected and non-infected (control) apples in ESI^+^ mode, and Figure 1b in ESI^−^ mode. In the ESI^+^ analysis, the model consisted of seven components (R2X = 0.684, Q2 = 0.379), with the first two components explaining 42% of the total variance (33% and 9%, respectively). In the case of ESI^−^, the seven-component model (R2X = 0.682, Q2 = 0.382) captured 41% of the variance in the two principal axes (29% for t [1] and 12% for t [2]). In both analyses, the control samples (at 25 °C and 4 °C) were separated from the infected apples, regardless of strain, inoculation site or incubation temperature. The control samples were consistently grouped at the right end of the score plots, where the highest positive values were found for principal component 1 (t [1]). This differential distribution confirms that infection is the predominant factor in the variation in the metabolic profile captured by the model. The ellipses shown in the PCA score plots represent the 95% confidence interval of the Hotelling T^2^ statistic. In both modes, the control samples at 25 °C and 4 °C showed group separation with the infected apples, independently from the strain, site of inoculation or incubation temperature.

Based on the trend observed in the PCA (Figure 1), a PLS-DA model was constructed to discriminate between infected samples (regardless of strain, inoculation zone or storage temperature) and non-infected samples used as controls. The initial models, constructed using all features with a *p*-value < 0.05, showed good predictive capacity, with Q2 values greater than 0.8 in both ionisation modes, 100% cross-validation and statistical significance confirmed by CV-ANOVA (*p*-value < 10^−9^) (Table S3). However, the relatively low R2X values suggest the high complexity of the model, associated with the large number of features included.

**Figure 2:**
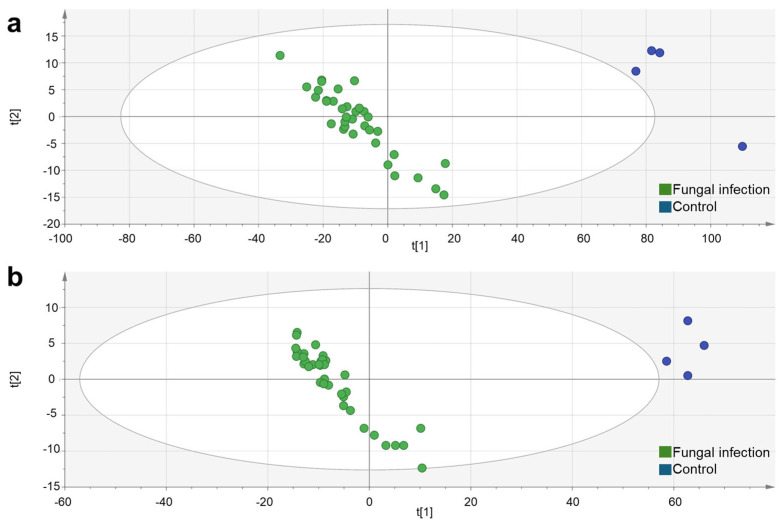
PLS-DA of metabolites of contaminated and uncontaminated apples. (**a**) ESI^+^ mode (R2X = 0.771, R2Y = 0.98 and Q2 = 0.935) and (**b**) ESI^−^ mode (R2X = 0.707, R2Y = 0.985 and Q2 = 0.931).

In order to improve the models, the number of features used to construct them was reduced based on VIP values. The models were then reconstructed using features with VIP > 1 (2837 features in positive mode and 4061 in negative mode). This reduction led to an improvement in the model parameters overall, particularly the R2X values, while maintaining high predictive power and statistical significance. Applying a more restrictive criterion (VIP > 1.5) reduced the number of features to 1400 in positive mode and 735 in negative mode. This resulted in highly robust models with Q2 values greater than 0.93, showing a clear improvement in the balance between descriptive and predictive capacity (Table S3). Furthermore, the validity and robustness of the model were confirmed by permutation tests, which were based on 50 random permutations per class (fungal infection samples and controls). The original R2 and Q2 values were consistently higher than those obtained in the permuted models, demonstrating that the observed high performance was not due to chance (Figures S1 and S2). These results suggest that a small subset of markers is sufficient for effectively discriminating between infected and uninfected samples. Figure 2 shows the PLS-DA score that discriminates between infected and uninfected samples using a reduced number of features (VIP > 1.5). The first latent variable (*x*-axis) distinguishes between both classes.

Next, the ability of the metabolomic profile to distinguish between samples based on the *Alternaria* strain responsible for the infection was assessed. To achieve this, new PLS-DA models were created using the data obtained in both ionisation modes. The modelling and validation strategy used was similar to that which was previously described. Initially, models were constructed using all features with a *p*-value lower than 0.05 and then optimised through progressive variable selection based on VIPs with thresholds greater than 1 and 1.5. The adjustment parameters, predictive capacity and statistical validation of the different models are summarised in Table S4.

The PLS-DA models demonstrated sufficient discriminatory capacity to distinguish between the *Alternaria* strains responsible for infection in both ionisation modes. In the ESI^−^ mode, all models were statistically significant, with high R2Y and Q2 values indicating excellent explanatory and predictive capacity. In ESI^+^ mode, using all features did not produce a statistically significant model. However, reducing the number of markers to the most relevant ones significantly improved the model’s performance, achieving high Q2 values and highly significant *p*-values. These results demonstrate the importance of feature selection in optimising discrimination between strains. To verify the accuracy of both models, permutation tests were conducted to ensure that the outcomes were not the result of chance.

**Figure 3:**
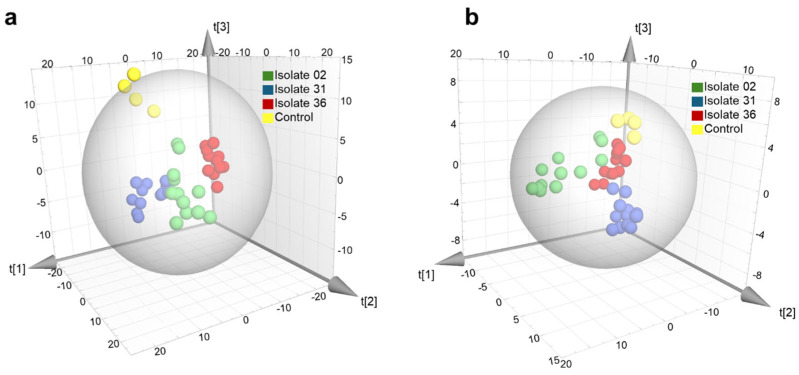
PLS-DA score to distinguish between *Alternaria* strain responsible for the infection. (**a**) ESI^+^ mode (R2X = 0.653, R2Y = 0.982 and Q2 = 0.852) and (**b**) ESI^−^ mode (R2X = 0.629, R2Y = 0.943 and Q2 = 0.819).

Figure 3 shows the PLS-DA score used to discriminate between the *Alternaria* strains responsible for apple infections using a reduced number of features (VIP > 1.5).

**Figure 4:**
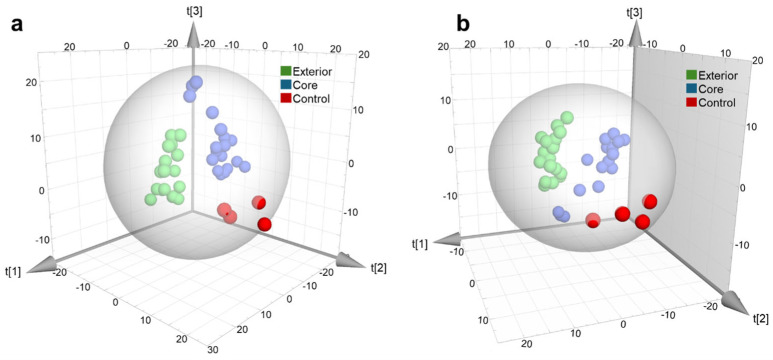
PLS-DA score to distinguish between the infected area of the apple. (**a**) ESI^+^ mode and (R2X = 0.545, R2Y = 0.987 and Q2 = 0.916) (**b**) ESI^−^ mode (R2X = 0.558, R2Y = 0.981 and Q2 = 0.917).

The next objective of the study was to determine whether the metabolic profile could be used to differentiate between infections that occurred on the exterior of the apple and those that occurred in the core. The results obtained for the different models are shown in Table S5. When considering all features, only the ESI^+^ model proved to be statistically significant, whereas no statistically significant *p*-value was observed in ESI^−s^ mode. Reducing the number of features (VIP > 1 and VIP > 1.5) considerably improved the performance of the models, with high Q2 values and significant *p*-values obtained in both the ESI^+^ and ESI^−^ models. These results suggest that the metabolomic profile can discriminate according to the infected area. However, this depends on the adequate refinement of the selected features since using the complete profile introduces noise that masks the relevant variables and leads to less robust models. Permutation tests reaffirmed the validity of both models and the corresponding PLS-DA scores using a reduced number of features (VIP > 1.5) are shown in Figure 4.

**Figure 5:**
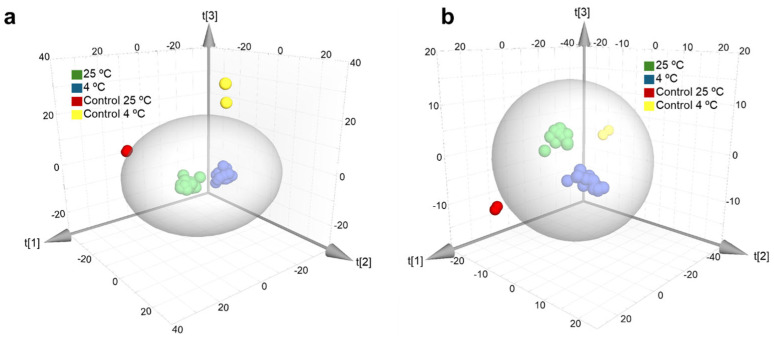
PLS-DA score to distinguish between the temperature at which the apples had been incubated. (**a**) ESI^+^ mode (R2X = 0.701, R2Y = 0.954 and Q2 = 0.867) and (**b**) ESI^−^ mode (R2X = 0.842, R2Y = 0.975 and Q2 = 0.941).

Then, the study examined whether the metabolomic profile could be discriminated based on the temperature at which the apples had been incubated. The results of the PLS-DA models using data acquired in ESI^+^ and ESI^−^ modes are presented in Table S6. The PLS-DA models exhibited robust behaviour, demonstrating high Q2 values and highly significant *p*-values in both ionisation modes, even when utilising all features. Subsequent variable reduction using VIPs maintained and even slightly improved the models’ predictive capacity without compromising their stability. These results highlight the significant impact of incubation temperature on the metabolomic profile of the samples, enabling clear discrimination. Permutation tests confirmed the validity of the models again, and the corresponding PLS-DA scores using a reduced number of features (VIP > 1.5) are shown in Figure 5.

Finally, the study examined whether the metabolomic profile could discriminate simultaneously between incubation temperature and the infected area (Table S7), *Alternaria* strain and infected area (Table S8), and *Alternaria* strain and incubation temperature (Table S9). The PLS-DA models generally exhibited lower discriminatory power than unifactorial analyses. In the combined analysis of incubation temperature and infected area (Table S7), statistically significant models with acceptable Q2 values were obtained in both ionisation modes only after selecting features with VIP > 1.5, indicating that it is possible to discriminate between these two factors together using a reduced number of features. In the analysis combining *Alternaria* strain and infected area (Table S8), the models showed limited performance with low Q2 values and a lack of statistical significance, even when the number of features was reduced. By contrast, combining the *Alternaria* strain and incubation temperature (Table S9) produced more robust models, particularly when the number of features was reduced (VIP > 1.5). This achieved acceptable Q2 values and significant *p*-values, highlighting an interaction between these factors in the metabolomic profile. Figures S3–S5 show the PLS-DA scores for these last three studies, using a reduced number of features (VIP > 1.5).

## 3. Discussion

Recent studies have confirmed that *Alternaria* contamination remains a relevant issue in apples, with several mycotoxins detected in apple products, highlighting the need for improved monitoring strategies throughout the production chain [3]. Moreover, previous results suggested the need for control strategies to prevent the presence of *Alternaria* mycotoxins in apple products [6]. The application of fungicides both in pre- and post-harvest stages does not provide an effective solution; moreover, fungicide resistance is increasing and new fungicide regulations are gaining more relevance [12,13]. There is a growing understanding that knowledge about the diversity in natural ecosystems can contribute to more sustainable crop production [14]. Since long-term storage increases the incidence of MC, an effective selection of raw material could prevent the incorporation of infected fruit into the process line. Several automated methods have been proposed for non-destructive detection of this disease. Hu et al. [15] recently developed a frequency domain diffuse optical tomography method for detecting underlying lesions of apple. The mouldy lesions not deeper than 20 mm from the peel were resolvable on the absorption images, but the model still presented several limitations. Yang and Yang [16] and Kadowaki et al. [17] developed non-destructive methods based on X-ray for detecting early stages of core rot in Japanese pear and suggested it could also be applied for apples. Methods based on transmittance spectroscopy were developed for the detection of single fruit with MC [18,19,20]; an online detection method based on visible and near-infrared spectroscopy full-transmittance spectra of MC apples was also developed [21]. All these methods are promising for fresh retail apples or to be applied by packhouses to prevent storage of fruit with early stages of MC. Nonetheless, none of them detect mycotoxin accumulation and therefore would not provide a comprehensive tool for processing industries.

The untargeted approach presented here, involving HRMS analysis, allowed detection of metabolomic features from the non-infected apples, the fungi, and their interaction. It clearly separated *Alternaria tenuissima*-infected from uninfected fruits, regardless of incubation temperature or place of inoculation, even in the conditions in which mycotoxin accumulation was lower (e.g., exterior infection, 9 months storage) [22]. This separation can be related to a group of metabolomic features produced by *Alternaria*’s interaction with the apples that are not present in the healthy apples incubated under any conditions. PC1 discriminated infected from not infected apples, and the conditions that favoured fungal secondary metabolites production were displaced to the left, taking negative values. Under these conditions, a more diverse metabolite production was detected, originating from the apple–fungal interaction. Figure 2 presents a supervised PLS-DA model in which class labels are incorporated to maximise discrimination between infected and control samples, resulting in an enhanced separation and demonstrating the strong predictive capacity of the metabolomic profile.

Although all strains used in this study were classified as *Alternaria tenuissima,* metabolic differences between apples infected with different strains were observed. Similarly, strain-dependent variability in secondary metabolite production has been previously reported for *Alternaria* [23,24]. In the present study, supervised PLS-DA modelling enabled the discrimination of strain-specific metabolic profiles, indicating differences in the metabolic responses elicited by distinct isolates.

The ability to discriminate between core and exterior infections only after refinement of the metabolomic feature set suggests that the location of the infection influences the metabolic profile in a subtle but consistent manner. MC represents a distinct ecological niche within the fruit, characterised by reduced oxygen availability, limited host defence responses, and prolonged moisture retention, all of which favour sustained fungal metabolism and toxin accumulation [25]. Our previous study showed that infections originating in the seed cavity are associated with higher and more persistent mycotoxin levels than surface infections, particularly during cold storage [22]. The necessity for variable reduction in the present study indicates that only a subset of metabolites is specifically associated with the infection site, while the full metabolomic profile contains substantial background variability related to apple tissue heterogeneity. These results support the concept that metabolomic discrimination of infection site is biologically meaningful but requires targeted marker selection to overcome matrix-driven noise.

Temperature emerged as one of the most influential factors shaping the metabolomic profile of infected apples, as demonstrated by the strong discriminatory performance of the PLS-DA models even before variable reduction. This observation is consistent with previous studies showing that storage temperature strongly modulates both fungal growth dynamics and secondary metabolite production in *Alternaria*-infected apples. Mao et al. [26] and Pavicich et al. [22] demonstrated that low-temperature storage does not suppress *Alternaria* activity but instead selects strains capable of sustained metabolic activity, including mycotoxin biosynthesis, over prolonged periods. In parallel, cold storage induces marked physiological and metabolic changes in apple tissue itself, including alterations in organic acids, phenolic compounds, and stress-related metabolites, which may further amplify differences between temperature conditions [27,28]. Therefore, the strong temperature-driven separation observed here likely reflects the combined effects of fungal metabolic adaptation and host stress responses during prolonged storage, reinforcing temperature as a critical determinant of the apple–*Alternaria* metabolomic interaction rather than a simple modulatory factor.

Differences observed between positive and negative ionisation modes across the various models highlight the complementary nature of these acquisition strategies. In particular, the superior performance of ESI^−^ in discriminating between *Alternaria* strains is consistent with the predominance of acidic secondary metabolites produced by *Alternaria* species, including dibenzo-α-pyrones, tetramic acids, and other polyketide-derived compounds, which are more efficiently ionised in negative mode. Previous chemical characterisations of *Alternaria* metabolite profiles have similarly reported improved detection and differentiation of strains when negative ionisation is employed [5]. Conversely, positive ionisation captured a broader range of host-derived metabolites, which may explain its stronger sensitivity to infection status but weaker initial strain discrimination. These findings underline the importance of dual-polarity acquisition in untargeted workflows and suggest that future method optimisation could prioritise negative ionisation for strain-level differentiation while retaining positive mode for comprehensive infection screening.

The reduced discriminatory power observed in multifactorial models combining *Alternaria* strain with infection site contrasts with the more robust performance obtained when strain was combined with incubation temperature. This suggests that temperature exerts a stronger and more systematic influence on the metabolomic outcome than infection location, potentially overriding strain-specific metabolic signatures in certain contexts.

The consistent improvement in model robustness following variable reduction highlights the relevance of focusing on a limited set of discriminatory markers rather than the full untargeted profile. High-complexity models based on thousands of features exhibited lower R2X values, reflecting the intrinsic variability of apple matrices and untargeted datasets. By contrast, models based on VIP-selected features achieved a more balanced descriptive and predictive performance, indicating that a relatively small subset of metabolomic features captures most of the biologically relevant information [29]. This has important implications for practical implementation, as it supports the future development of targeted or semi-targeted screening methods that retain discriminatory power while reducing analytical complexity, data processing time, and cost.

The present study was designed as a feature-based metabolomic screening approach aimed at evaluating the discriminatory potential of untargeted LC-HRMS fingerprints rather than performing structural metabolite identification. The variables selected through VIP-based reduction correspond to mass–retention time features detected in untargeted mode and were not subjected to compound annotation beyond feature level. Because these discriminant features are model-dependent and differ according to the specific comparison performed (infection status, strain, infection site, or storage temperature), they cannot yet be interpreted as validated biomarkers of *A. tenuissima* infection. Structural elucidation and confirmation of the most relevant signals would require additional targeted MS/MS experiments, spectral library matching, and reference standard verification. Such work represents a logical next step toward biological interpretation and the development of simplified targeted screening methods but that was beyond the scope of the present proof-of-concept study. Although the metabolomic features responsible for discrimination were not structurally identified in the present study, the consistent classification performance indicates that the observed patterns are not random but reflect reproducible metabolic responses. Untargeted metabolomics has repeatedly shown that robust discrimination can be achieved even in the absence of full compound identification, particularly in complex plant–pathogen systems [10]. Nevertheless, the construction of a database focused on *Alternaria* secondary metabolites and apple defence compounds would significantly enhance interpretability and facilitate biomarker validation. Similar untargeted-to-targeted transitions have enabled the identification of early infection markers in other fruit–fungus systems and represent a logical next step for translating the present findings into applied control strategies [11,30].

This HRMS method sets the basis for further development of targeted analysis to prevent the presence of *Alternaria* mycotoxins in the final products. Moreover, this HRMS-based approach could be used by apple-processing industries as a screening strategy to flag batches showing metabolomic signatures associated with *Alternaria* infection and an increased risk of mycotoxin presence in the final products. After raw material is incorporated in the process line, the first step consists of grinding the fruit, from which a representative sample could be analysed. Within a 15 min chromatographic run, batches exhibiting infection-related metabolomic signatures could be identified. These flagged batches could then be subjected to mycotoxin quantification to support decisions regarding their subsequent processing or destination. When low levels of *Alternaria* toxins are detected, the batch could be destined for the production of clarified apple products, since clarification reduces their concentration [31]. If high levels of mycotoxin are found in contaminated batches, then an alternative use as compost should be evaluated [32]. Additionally, detoxification methods, such as UV radiation, which proved to degrade patulin in apple juice [33], or treatments with fungal enzymes that were effective in the degradation of other mycotoxins [34], could be investigated. The proposed workflow represents a proof-of-concept screening strategy rather than a validated industrial method and would require further validation across different apple cultivars, harvest seasons, production batches, and processing conditions prior to routine implementation.

## 4. Conclusions

This work presents the first HRMS models to detect apple infection with *Alternaria tenuissima*. This study demonstrates that untargeted LC-HRMS metabolomic fingerprinting enables robust discrimination between *Alternaria tenuissima*-infected and non-infected apples under retail and long-term storage conditions. The chemometric models showed high predictive performance and were capable of distinguishing infection status independently of strain, infection site, and incubation temperature. These findings support the feasibility of applying feature-based metabolomic signatures as a screening strategy for detecting infected fruit before processing.

Although the present work focused on *A. tenuissima* and operates at the level of metabolomic features rather than structurally identified compounds, the results provide a solid foundation for future targeted investigations. Further studies aimed at metabolite annotation, validation across additional cultivars and *Alternaria* species, and the development of simplified screening workflows will be necessary to translate this approach into routine food safety applications.

## 5. Materials and Methods

### 5.1. Sample Preparation

The study used healthy Red Delicious apples, which were subjected to artificial infection with *Alternaria* strains. Three strains previously isolated from apples and classified as *A. tenuissima* were selected based on their production of secondary metabolites [24]. Isolate 02 had been isolated from the surface of the fruit, while Isolates 31 and 36 were from the core. These strains (isolates ID: 02, 31 and 36) were used to inoculate healthy fruits of the selected variety.

The apples, which were purchased from a local market, were surface-disinfected using 70% ethanol for 10 min. Inoculation was performed both on the surface and inside the fruit in separate trials. For surface inoculation, a shallow incision measuring 1 × 1 cm was made using a sterile scalpel. The fungal suspensions were prepared in an aqueous solution of Tween 80 (0.05%), with the final concentration adjusted to 10^5^ spores/mL. At each inoculation point, 1 µL of the relevant suspension was applied using a sterile, calibrated loop. The apples were then incubated at 25 °C for one month and at 4 °C for nine months to simulate market and storage conditions, respectively. All tests were performed in triplicate, with each apple incubated individually in a sterile bag. This resulted in a total of 36 contaminated apple samples. Non-inoculated apples were also included as negative controls to obtain blank samples. These samples were incubated under the same conditions: two at 25 °C for one month and two at 4 °C for nine months.

### 5.2. Extraction

The extraction solvent and conditions were selected based on previous in-house experience with apple matrices and *Alternaria* secondary metabolites, as well as on commonly applied dilute-and-shoot extraction approaches used in untargeted LC-HRMS metabolomics of food samples. Briefly, apportion of 2.0000 ± 0.002 g of each fruit was placed in an extraction tube and extracted with 10 mL of a mixture of methanol, ultrapure water, and acetic acid (MeOH/H_2_O/AA, 79:20:1, *v*/*v*/*v*) for 30 min in a shaker. The samples where then centrifuged at 4000× *g* for 15 min and a 1 mL aliquot was taken from the supernatant and evaporated till dryness under a N_2_ steam. Samples were resuspended in the injection solvent H_2_O/ACN (70:30, *v*/*v*) and filtered through PTFE filters.

### 5.3. UPLC/HRMS Analysis

UPLC/HRMS conditions were adapted from a reported method [24] in combination with previous in-house experiments [35]. An aliquot of 5 µL was injected into an ACQUITY UPLC system coupled to a Synapt G2–Si High-Definition instrument, a hybrid quadrupole orthogonal acceleration time of flight equipped with travelling wave ion mobility separation mass spectrometer (Waters Corporation, Milford, MA, USA). The column was a HSS T3 (1.8 µm, 2.1 × 100 mm) held at 40 °C and samples were maintained at 10 °C (Waters Corporation). A linear gradient elution programme with solvent A (ultrapure water: 20 mmol L^−1^ formic acid) and B (ACN: 20 mmol L^−1^ formic acid) was applied with a flow rate of 0.35 mL/min as follows: 90% A and 10% B for 0.5 min; an increase to 100% B from 0.5 to 10.0 min; 100% B maintained from 10.0 to 13.0 min, with a direct back to 90% A from 13.0 to 13.1 min, and maintaining starting conditions from 13.1 to 15 min. Ultrapure water, acetonitrile and formic acid were LC-MS grade and obtained from Sigma Aldrich (Bornem, Belgium). The instrument was operated in resolution mode and calibration was done with sodium formate clusters. Leucine enkephalin was used as lock mass for mass correction with a scan time of 0.1 s and a frequency of 20 s. Data type was continuum and acquired in MS^E^ mode on ESI^+^ and ESI^−^ in separate runs in the scan range *m/z* 50 to 1200 Da. Mass spectrometry parameters were as follows: capillary voltage 2.8 kV; sample cone voltage 40 V; source offset 80 °C; source temperature 130 °C; desolvation gas flow 800 L/h at a temperature of 550 °C; cone gas flow 50 L/h. Nitrogen was employed as desolvation and cone gas at a pressure of 6.5 bar. Argon was used as the collision gas at a 9.28 × 10^−3^ mbar. Ion fragmentation was performed using a mass-dependent collision energy ramp implemented on the instrument. The ramp was defined by two anchor points at *m/z* 50 and *m/z* 1200. At the low-mass anchor (*m/z* 50), the collision energy was set to range from 11 to 13 V, while at the high-mass anchor (*m/z* 1200), it ranged from 50 to 120 V. For precursor ions with intermediate *m/z* values, collision energies were automatically computed by linear interpolation between these limits, ensuring appropriate energy scaling across the entire mass range.

### 5.4. Data Processing

Progenesis QI (Waters Corporation) was used to analyse the large amount of data generated. The adduct ions [M+H]^+^, [M+Na]^+^, [M+NH_4_]^+^, [M+H-H_2_O]^+^ were selected in the positive mode and [M-H]^−^, and [M+HCOO]^-^ in the negative mode. Then, pre-processing by retention time alignment and peak picking, which involved the information of retention time, *m/z*, and peak area, was done. Composite ion maps were obtained, with the statistical tools included in Progenesis QI (Waters Corporation, Milford, MA, USA); metabolomic features were filtered according to ANOVA *p*-value < 0.05 prior to principal component analysis.

The chemometric models were constructed using SIMCA software (version 14.1, Umetrics, Sartorius Stedim Biotech AB, Umeå, Sweden), with the data obtained in positive and negative ionisation modes analysed separately. Prior to model creation, the data were normalised using logarithmic transformation due to their non-normal distribution. Auto-scaling was then applied using the unit variance (UV) method to prevent characteristics with a large area from dominating the model and over those with a smaller area.

Partial least squares discriminant analysis (PLS-DA) models were evaluated using the parameters R2X, R2Y and Q2, whose values ranged from 0 to 1. R^2^ values close to one indicate an adequate descriptive capacity of the model, while high Q2 values indicate good predictive performance. According to the established criteria, models with Q2 values greater than 0.5 were considered satisfactory [36]. Additionally, the cross-validation percentage was examined, and permutation tests were performed using 50 random permutations. The statistical significance of the models was evaluated by analysing the variance of the predictive residuals obtained by cross-validation (CV-ANOVA), with a *p*-value threshold of <0.05 used to confirm statistical validity [37].

In order to optimise the models and reduce the number of features included, the variable importance values (VIP) derived from the initial models were analysed. Based on this analysis, the models were reconstructed using only the features with VIP > 1 [38]. Alternative models were also created using a more restrictive threshold of VIP > 1.5.

## Data Availability

The original contributions presented in this study are included in the article and Supplementary Materials. Further inquiries can be directed to the corresponding author.

## Supplementary Materials

The following supporting information can be downloaded at https://www.mdpi.com/article/10.3390/toxins18040159/s1, Table S1. Selected metabolomic features used for downstream chemometric analysis in ESI^+^ mode. Table S2. Selected metabolomic features used for downstream chemometric analysis in ESI^−^ mode. Table S3. PLS-DA evaluation parameters to distinguish between infected and non-infected samples; Figure S1. Permutation plots for the PLS-DA model using ESI^+^ data. (a) Fungi infection and (b) Control; Figure S2. Permutation plots for the PLS-DA model using ESI^−^ data. (a) Fungi infection and (b) Control; Table S4. PLS-DA evaluation parameters to distinguish between *Alternaria* strains responsible for the infection; Table S5. PLS-DA evaluation parameters for differentiation according to the infected area of the apple; Table S6. PLS-DA evaluation parameters for differentiation according to the temperature at which the apples had been incubated; Table S7. PLS-DA evaluation parameters for differentiation according incubation temperature and infected area; Table S8. PLS-DA evaluation parameters for differentiation according to the *Alternaria* strains responsible for the infection and the infected area; Table S9. PLS-DA evaluation parameters for differentiation according to the *Alternaria* strains responsible for the infection and the temperature at which the apples had been incubated; Figure S3. PLS-DA score to distinguish between the temperature at which the apples had been incubated and infected area. (a) ESI^+^ mode and (b) ESI^−^ mode; Figure S4. PLS-DA score to distinguish between the *Alternaria* strain and infected area. (a) ESI^+^ mode and (b) ESI^−^ mode; Figure S5. PLS-DA score to distinguish between the *Alternaria* strain and the temperature at which the apples had been incubated. (a) ESI^+^ mode and (b) ESI^−^ mode.
