# Supplementary material for: Untargeted LC-HRMS Metabolomics for the Detection of Alternaria-Infected Apples Under Retail and Storage Conditions
Source: Toxins (Basel). 2026 Mar 27;18(4):159. doi: 10.3390/toxins18040159 (PMC13119880; doi:10.3390/toxins18040159)
Supplement: Supplementary file 1 [file toxins-18-00159-s001.zip › Supplementary material.pdf]

Supplementary Materials: Untargeted LC-HRMS Metabolomics for the Detection of *Alternaria*-Infected Apples Under Retail and Storage Conditions

María Agustina Pavicich , Claudia Giménez-Campillo, José Diana Di Mavungu, Sarah De Saeger and Andrea Patriarca

Table S3. PLS-DA evaluation parameters to distinguish between infected and non-infected samples.

Mode	Features	Components	R2X	R2Y	Q2	CV	p-value
ESI^+	9,308	4	0.407	0.994	0.871	100%	3×10^{-11}
ESI^-	10,505	4	0.485	0.995	0.894	100%	8×10^{-10}
ESI^+	2,837	3	0.684	0.984	0.91	100%	6×10^{-13}
ESI^-	4,061	3	0.588	0.99	0.936	100%	2×10^{-13}
ESI^+	1,400	2	0.771	0.98	0.935	100%	3×10^{-19}
ESI^-	735	2	0.707	0.985	0.931	100%	7×10^{-18}

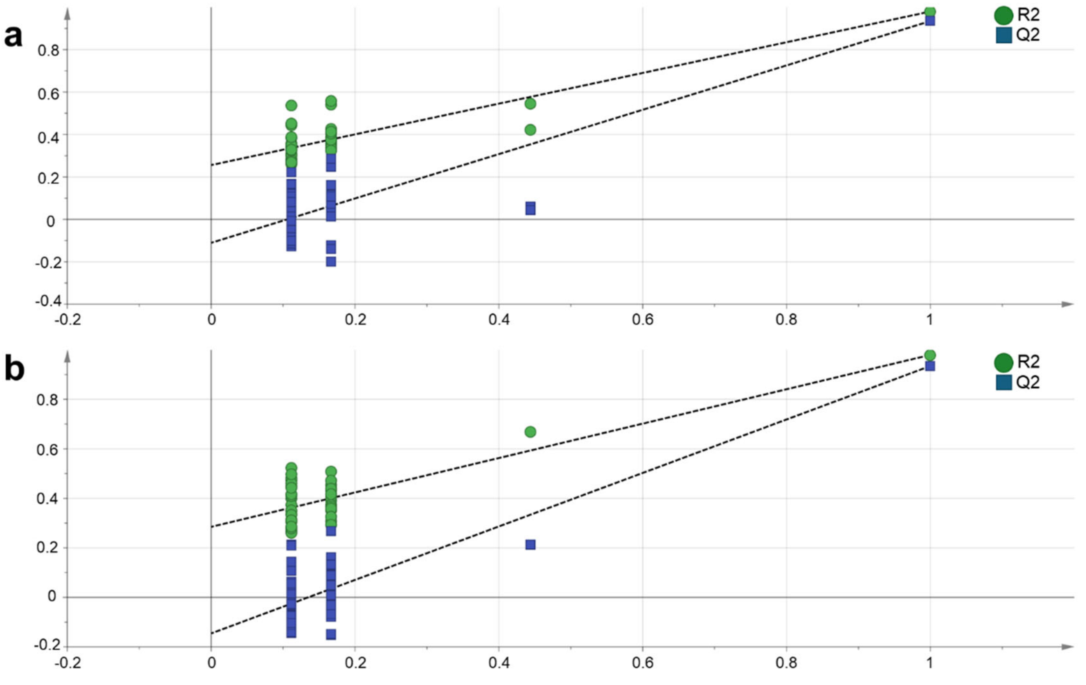

Figure S1. Permutation plots for the PLS-DA model using ESI^+ data. (a) Fungi infection and (b) Control.

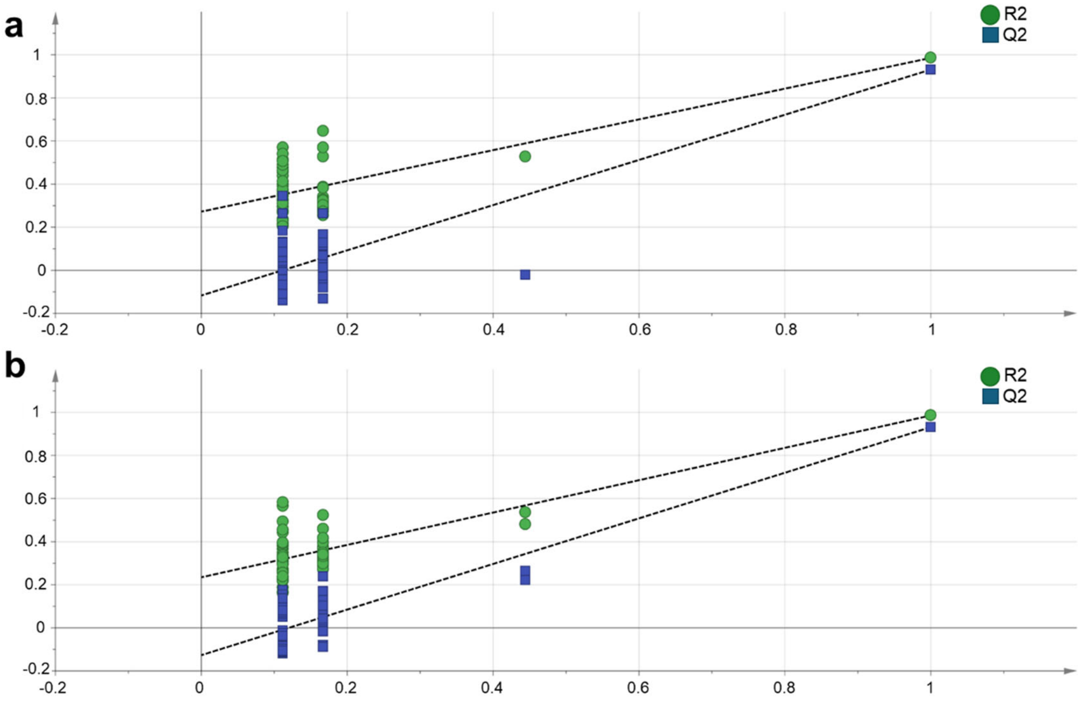

Figure S2. Permutation plots for the PLS-DA model using ESI$^-$ data. (a) Fungi infection and (b) Control.

Table S4. PLS-DA evaluation parameters to distinguish between *Alternaria* strains responsible for the infection.

Mode	Features	Components	R2X	R2Y	Q2	CV	p-value
ESI $^+$	9,308	9	0.702	0.986	0.716	100%	0.482
ESI $^-$	10,505	9	0.689	0.981	0.819	100%	0.0001
ESI $^+$	3,506	8	0.656	0.989	0.886	100%	2×10^{-6}
ESI $^-$	4,256	8	0.674	0.981	0.866	100%	3×10^{-10}
ESI $^+$	295	8	0.653	0.982	0.852	100%	5×10^{-11}
ESI $^-$	143	6	0.629	0.943	0.819	100%	5×10^{-18}

Table S5. PLS-DA evaluation parameters for differentiation according to the infected area of the apple.

Mode	Features	Components	R2X	R2Y	Q2	CV	p-value
ESI $^+$	9,308	6	0.598	0.979	0.761	100%	0.008
ESI $^-$	10,505	6	0.578	0.976	0.760	100%	0.949
ESI $^+$	3,265	6	0.631	0.986	0.906	100%	1×10^{-11}
ESI $^-$	4,256	6	0.650	0.982	0.829	100%	0.02
ESI $^+$	444	6	0.545	0.987	0.916	100%	5×10^{-15}
ESI $^-$	507	5	0.558	0.981	0.917	100%	8×10^{-17}

Table S6. PLS-DA evaluation parameters for differentiation according to the temperature at which the apples had been incubated.

Mode	Features	Components	R2X	R2Y	Q2	CV	p-value
ESI $^+$	9,308	7	0.647	0.989	0.890	100%	1×10^{-10}
ESI $^-$	10,505	7	0.638	0.983	0.833	100%	5×10^{-8}
ESI $^+$	3,768	4	0.604	0.953	0.826	100%	2×10^{-12}
ESI $^-$	4,446	4	0.617	0.940	0.819	100%	5×10^{-16}
ESI $^+$	354	3	0.701	0.954	0.867	100%	1×10^{-12}
ESI $^-$	205	4	0.842	0.975	0.941	100%	1×10^{-21}

Table S7. PLS-DA evaluation parameters for differentiation according incubation temperature and infected area.

Mode	Features	Components	R2X	R2Y	Q2	CV	p-value
ESI $^+$	9,308	8	0.667	0.952	0.650	100%	0.372
ESI $^-$	10,505	7	0.626	0.908	0.398	100%	0.982
ESI $^+$	3846	7	0.619	0.946	0.750	100%	0.043
ESI $^-$	4291	7	0.531	0.902	0.401	100%	0.999
ESI $^+$	164	7	0.640	0.925	0.698	100%	2×10^{-8}
ESI $^-$	241	6	0.700	0.907	0.707	100%	0.001

Table S8. PLS-DA evaluation parameters for differentiation according to the *Alternaria* strains responsible for the infection and the infected area.

Mode	Features	Components	R2X	R2Y	Q2	CV	p-value
ESI $^+$	9,308	4	0.479	0.496	0.229	72.5%	0.995
ESI $^-$	10,505	5	0.550	0.524	0.153	87.5%	1
ESI $^+$	3660	4	0.431	0.521	0.330	62.5%	0.185
ESI $^-$	5007	4	0.605	0.445	0.106	67.5%	1
ESI $^+$	427	4	0.437	0.545	0.408	80%	0.0002
ESI $^-$	252	4	0.799	0.425	0.262	67.5%	0.922

Table S9. PLS-DA evaluation parameters for differentiation according to the *Alternaria* strains responsible for the infection and the temperature at which the apples had been incubated.

Mode	Features	Components	R2X	R2Y	Q2	CV	p-value
ESI $^+$	9,308	9	0.697	0.942	0.649	100%	0.255
ESI $^-$	10,505	9	0.691	0.931	0.640	100%	0.030
ESI $^+$	3866	9	0.662	0.959	0.743	100%	0.067
ESI $^-$	4390	9	0.600	0.902	0.500	100%	0.91
ESI $^+$	166	7	0.638	0.907	0.564	100%	0.01
ESI $^-$	182	9	0.703	0.941	0.706	100%	3×10^{-10}

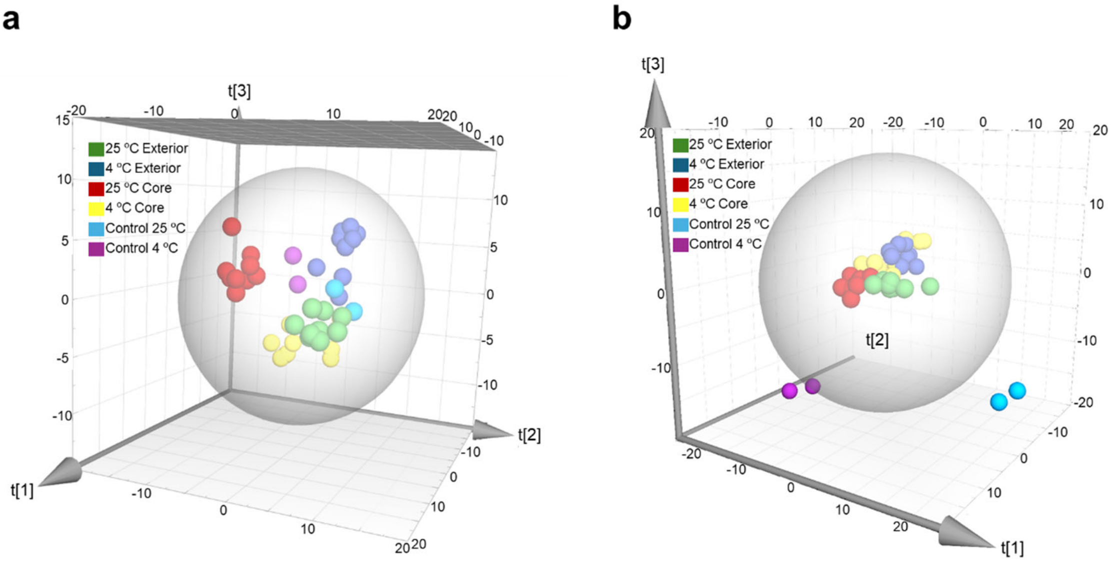

Figure S3. PLS-DA score to distinguish between the temperature at which the apples had been incubated and infected area. a) ESI^+ mode (R2X = 0.640, R2Y = 0.925 and Q2 = 0.698) and b) ESI^- mode (R2X = 0.700, R2Y = 0.907 and Q2 = 0.707).

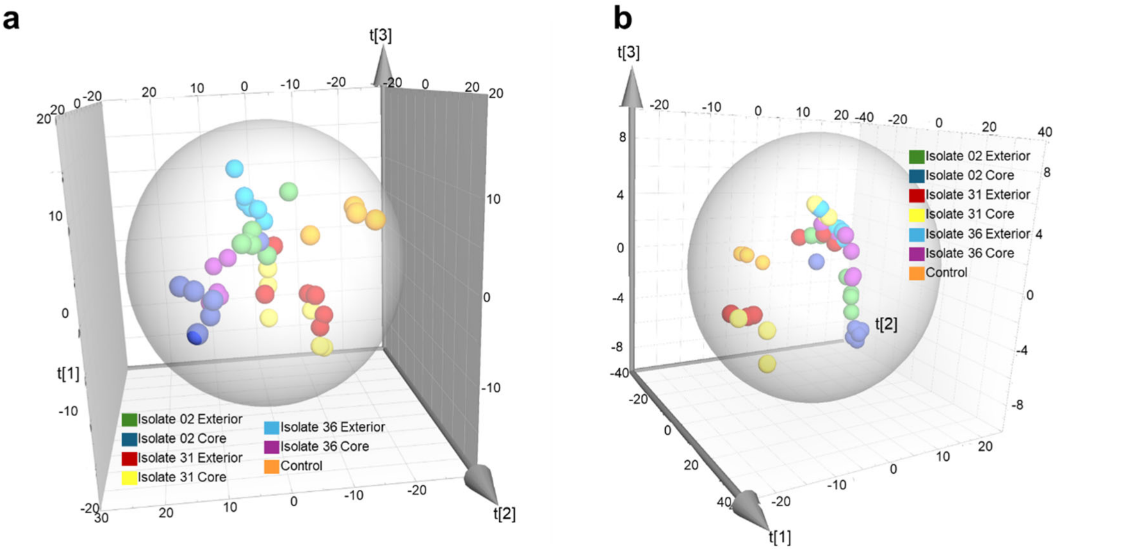

Figure S4. PLS-DA score to distinguish between the *Alternaria* strain and infected area. a) ESI^+ mode (R2X = 0.437, R2Y = 0.545 and Q2 = 0.408) and b) ESI^- mode (R2X = 0.799, R2Y = 0.425 and Q2 = 0.262).

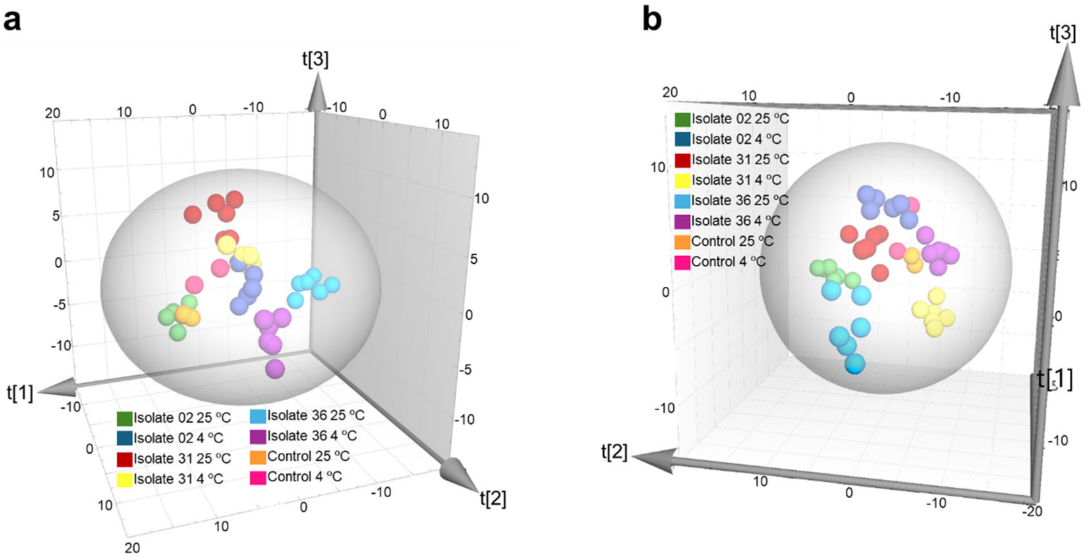

Figure S5. PLS-DA score to distinguish between the *Alternaria* strain and the temperature at which the apples had been incubated. a) ESI^+ mode (R2X = 0.638, R2Y = 0.907 and Q2 = 0.564) and b) ESI^- mode (R2X = 0.703, R2Y = 0.941 and Q2 = 0.706).
